# X-ray zooming optics for analyzer-based multi-contrast computed tomography

**DOI:** 10.1107/S1600577522001412

**Published:** 2022-03-15

**Authors:** Keiichi Hirano, Hiroshi Sugiyama, Ryutaro Nishimura, Daisuke Wakabayashi, Yoshio Suzuki, Noriyuki Igarashi, Nobumasa Funamori

**Affiliations:** a Photon Factory, Institute of Materials Structure Science, High Energy Accelerator Research Organization, 1-1 Oho, Tsukuba, Ibaraki 305-0801, Japan

**Keywords:** analyzer-based imaging, X-ray Bragg magnifier, computed tomography

## Abstract

An X-ray analyzer-based optics with a zoom function is proposed for observing various samples with apparent-absorption contrast, phase contrast and scattering contrast. The proposed X-ray optics consists of a collimator crystal and an analyzer crystal arranged in a nondispersive (+, −) geometry with a sample placed between them. For the implementation of the zoom function, an asymmetrically cut crystal in the rotated-inclined geometry was used for the analyzer. Proof-of-principle experiments were performed at the vertical wiggler beamline BL-14B of the Photon Factory. First, the magnification was set to 1×, and then it was zoomed into the optimal magnification (10×). At these magnifications, tri-modal contrast cross-sectional images of a sample were obtained by computed tomography. It was confirmed that the image quality at 10× was superior to that at 1×. This achievement opens up new possibilities for observing an entire sample or regions of interest within a sample at optimal magnification, and is expected to be useful for materials science, condensed matter physics, archeology and biomedical science.

## Abstract 2

An X-ray analyzer-based optics with a zoom function is proposed for observing various samples with apparent-absorption contrast, phase contrast and scattering contrast. Proof-of-principle experiments were performed and tri-modal contrast cross-sectional images of a sample were obtained at 1× and 10× magnification.

## 1. Introduction

X-ray analyzer-based imaging (ABI) is a powerful method for nondestructive observation of the inner structures of a wide variety of specimens (Davis *et al.*, 1995 ▸; Chapman *et al.*, 1997 ▸). One of the most striking features of ABI is that it can produce not only apparent-absorption contrast and phase contrast images but also scattering contrast images from a set of recorded images (Pagot *et al.*, 2003 ▸; Rigon *et al.*, 2007 ▸). Thanks to this advantage, ABI has been widely used for research in such fields as materials science, condensed matter physics, archeology and biomedical science.

Another advantage of ABI is that the magnification of a sample image can be realized with an asymmetrically cut analyzer crystal. This type of magnifying optics has been used for synchrotron radiation (Modregger *et al.*, 2006 ▸; Hönnicke & Cusatis, 2007 ▸; Hirano, 2011*a* ▸,*b* ▸) as well as for laboratory X-ray sources (Vagovič *et al.*, 2015 ▸). There is, however, a limitation in that the magnification factor is fixed at a certain value depending on the experimental conditions. Because of this lack of tunability of the magnification factor, the observation of an entire sample or region of interest (ROI) within a sample has not necessarily been performed under the optimal conditions. To solve this problem, we pursued the possibility of adding a zoom function to the analyzer. One of the advantages of this optical approach is that it allows us to use a variety of high-performance X-ray area sensors. For example, the combination of the zooming optics with direct-conversion X-ray cameras such as Pilatus (Broennimann *et al.*, 2006 ▸; Kraft *et al.*, 2009 ▸), SOIPIX (Arai *et al.*, 2011 ▸) and Medipix (Llopart *et al.*, 2002 ▸; Ballabriga *et al.*, 2018 ▸) will open up new possibilities for high-speed, high-sensitivity and wide-dynamic-range imaging. Toward this end, we developed an X-ray Bragg magnifier with a zoom function (Hirano *et al.*, 2014 ▸). In this paper, we propose the incorporation of this optical element into ABI to realize an X-ray zooming optical system for observing not only apparent-absorption contrast and phase contrast images but also scattering contrast images.

## 2. Principle of analyzer-based multi-contrast zooming computed tomography

### 2.1. X-ray Bragg magnifier with a zoom function

Conventional X-ray Bragg magnifiers are based on the asymmetric diffraction of X-rays at a nearly perfect crystal such as silicon, diamond and germanium (Kohra, 1972 ▸; Boettinger *et al.*, 1979 ▸; Takagi *et al.*, 1985 ▸; Kuriyama *et al.*, 1990 ▸). In this geometry, the diffracted X-rays are either expanded or compressed in the direction parallel to the plane of diffraction defined by the incident beam and the diffracted beam. In general, asymmetric diffraction is characterized by the asymmetry factor, *b*, given by

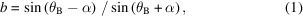

where θ_B_ is the Bragg angle and α is the angle between the crystal surface and the diffracting lattice planes. Here, the sign of the asymmetric angle, α, is positive in the expansion mode and negative in the compression mode. The magnification factor, *M*, is given by

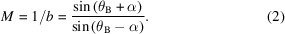

**Figure 1:**
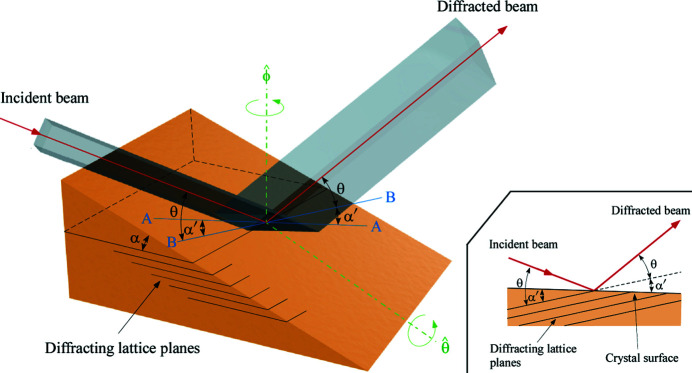
Overview of the X-ray Bragg magnifier with a zoom function. The orientation of the crystal is adjusted with the 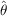 -axis and 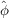 -axis. The 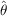 -axis is perpendicular to the plane of diffraction defined by the incident beam and the diffracted beam. The 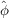 -axis is perpendicular to the diffracting lattice planes. Diffracted X-rays are either expanded or compressed in the direction parallel to the plane of diffraction. Line A–A is the intersection between the crystal surface and the plane of diffraction. Line B–B is the intersection between the plane of diffraction and one of the diffracting lattice planes. The effective asymmetric angle, α′, is defined as the angle between line A–A and line B–B. The inset shows the X-ray diffraction geometry in the plane of diffraction.

This equation shows that the magnification factor of the conventional Bragg magnifier is fixed once θ_B_ and α are given. To achieve a zoom function, we introduced a new rotation axis, 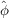 , as shown in Fig. 1 ▸. Since the 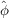 -axis is perpendicular to the diffracting lattice planes, the ϕ angle can be changed while maintaining the Bragg diffraction condition. This diffraction geometry is known as the rotated-inclined geometry (Smither & Fernandez, 1994 ▸) and is widely used for high-heat-load monochromators at the X-ray beamlines of synchrotron radiation facilities (Uruga *et al.*, 1995 ▸; Yabashi *et al.*, 1999 ▸). A special case of this geometry (ϕ = 0°) is known as the inclined geometry (Hrdý, 1992 ▸; Macrander *et al.*, 1992 ▸; Khounsary, 1992 ▸; Lee *et al.*, 1992 ▸; Hrdý & Pacherová, 1993 ▸), where the magnification factor is fixed at 1×. For ϕ = +90° (ϕ = −90°), we have the conventional asymmetric situation at a low (high) incident angle with the fixed magnification factor given by equation (2).

The magnification factor, *M*, of our Bragg magnifier with the zoom function depends on the effective asymmetric angle, α′. Line A–A in Fig. 1 ▸ is the intersection between the crystal surface and the plane of diffraction. Line B–B is the intersection between the plane of diffraction and one of the diffracting lattice planes. The effective asymmetric angle, α′, is defined as the angle between line A–A and line B–B and is given by

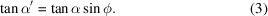

The asymmetry factor and the magnification factor are given by

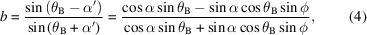




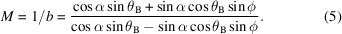

**Figure 2:**
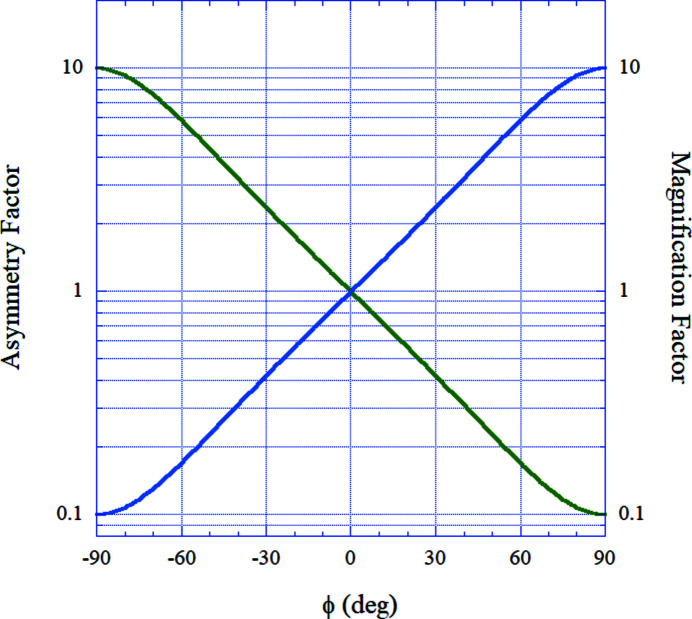
Calculated asymmetry factor (green line) and magnification factor (blue line) for a Si(220) crystal with an asymmetric angle of 14° at a wavelength of 0.112 nm.

Fig. 2 ▸ shows an example of the calculated asymmetry factor (green line) and magnification factor (blue line) for a Si(220) crystal with an asymmetric angle of 14° at a wavelength of 0.112 nm. With the increase in the azimuthal angle, ϕ, from −90° to +90°, the magnification factor *M*, contiuously varies from 0.1 to 10. This result clearly shows that we can continuously control *M* through the azimuthal angle, ϕ.

Another important property of the asymmetric diffraction is the angular width of the intrinsic rocking curve which is given by

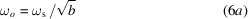

for the incident beam and

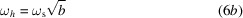

for the outgoing beam. Here, ω_s_ is the angular width of the intrinsic rocking curve for the symmetric diffraction (α = 0°). The penetration of X-rays into the crystal is also important because it causes a geometrical blur. This effect is estimated by

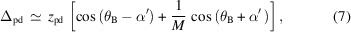

where *z*_pd_ is the penetration depth of the X-rays. Both ω_s_ and *z*_pd_ can be calculated by the dynamical theory of X-ray diffraction (Zachariasen, 1945 ▸; Batterman & Cole, 1964 ▸; Ishikawa & Kohra, 1991 ▸; Authier, 2001 ▸).

### 2.2. X-ray multi-contrast zooming optics for ABI

**Figure 3:**
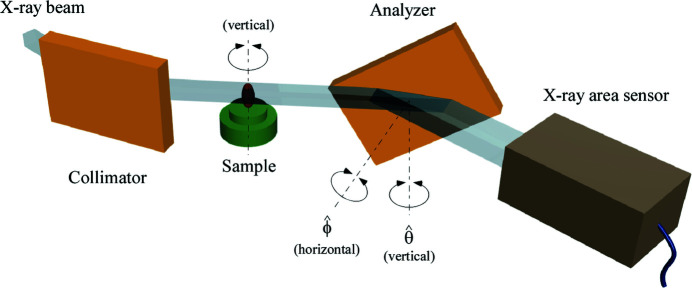
X-ray multi-contrast zooming optics for ABI. The optics consists of a collimator crystal and an analyzer crystal arranged in a nondispersive (+, −) geometry with a sample placed between them. The sample is mounted on a rotation stage for acquiring the CT data set. The analyzer crystal plays an essential role not only as an angular filter but also as a Bragg magnifier with a zoom function.

The X-ray optics of ABI usually consists of a collimator crystal and an analyzer crystal arranged in a nondispersive (+, −) geometry with a sample placed between them. To add a zooming capability to the conventional ABI optics, an X-ray Bragg magnifier with a zoom function was incorporated into the analyzer. Fig. 3 ▸ schematically shows our ABI optics with a zoom function. Incident monochromatic X-rays are diffracted by the collimator and pass through the sample where absorption, refraction and scattering take place. The X-rays are then diffracted by the analyzer and recorded with an X-ray area sensor. Here, the analyzer plays an essential role not only as an angular filter but also as a Bragg magnifier with a zoom function.

The angular resolution of the optics depends on the shape of the rocking curve of the analyzer. From the DuMond diagram (DuMond, 1937 ▸), the angular width of the rocking curve of the analyzer is given by

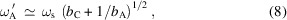

where *b*_C_ and *b*_A_ are the asymmetry factors of the collimator and the analyzer, respectively.

The spatial resolution of this optical system depends on the spatial resolution of the area sensor, Δ_sensor_. This effect is estimated by

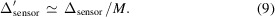

The spatial resolution also depends on the penumbral blurring given by

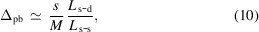

where *s* is the source size, *L*_s–s_ is the source-to-sample distance, and *L*_s–d_ is the sample-to-detector distance. Under conditions where geometrical optics is valid, the spatial resolution of the entire optical system can be estimated by

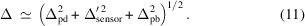




Multi-contrast images of the sample can be obtained by the analyzer scanning method (Pagot *et al.*, 2003 ▸). In this method, it is necessary to take a series of *N* images along the rocking curve (RC) of the analyzer to obtain the individual RC at each pixel of the area sensor. A series of images taken with the sample gives an object RC for each pixel, whereas that without the sample gives a reference RC. In the image analysis, the zero-, first- and second-order moments are calculated according to the following equation,

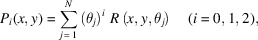

where *R* is the intensity in a pixel and θ_*j*_ is the angle of the analyzer. *x* (*y*) is the abscissa (ordinate) coordinate of the detector pixel. For simplicity, we assume that the *x*-axis (*y*-axis) is parallel (perpendicular) to the plane of diffraction. Each moment has the following physical meaning: (i) the zero-order moment, *P*_0_, gives the integrated intensity, (ii) the first-order moment, *P*_1_, gives the center of mass θ_cm_ = *P*_1_/*P*_0_, and (iii) the second-order moment, *P*_2_, gives the standard deviation σ = 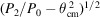 . From these moments, multi-contrast images can be calculated. First, the apparent-absorption contrast image is obtained by estimating the transmittance of the X-rays, which is defined as the ratio of the integrated intensities calculated for the object RC to that for the reference RC, and is given by

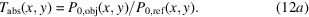




Second, the refraction contrast image is obtained by calculating the angular displacement of the center of mass of the object RC with respect to that of the reference RC, and is given by

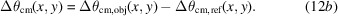

Since the refraction angle is linked to the gradient of the phase, the phase contrast image is given by

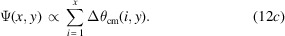

Because the sensitivity of the phase contrast is much higher than that of the absorption contrast for light elements, the phase contrast is especially suitable for observation of biological soft tissues and polymeric objects.

Third, the ultra-small-angle X-ray scattering contrast (USAXS contrast) image is obtained by estimating the difference in the standard deviation between the object RC and the reference RC, and is given by

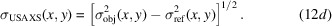

The USAXS contrast reflects the textural features of the sample and reveals information about the sample on a sub-pixel scale.

Thus, we can calculate the apparent-absorption, refraction, phase and USAXS contrast projection images of the sample from a series of images obtained by scanning the analyzer through the Bragg diffraction condition. It is worth noting that, in the analyzer scanning method, flat-field correction is not necessary because the normalization process is inherent in the algorithm, as shown in equations (12*a*)–(12*d*).

Three-dimensional images of a sample are obtained by computed tomography (CT). In our optics, the rotation axis of the sample is set to be perpendicular to the plane of diffraction of the analyzer. This is a common choice in X-ray differential phase contrast tomography where there is interest in obtaining phase sensitivity in the reconstructed slices that are perpendicular to the rotation axis. Magnified reconstructed slices are also obtained since the magnification is in the direction perpendicular to the rotation axis.

## 3. Experimental and results

The proof-of-principle experiments of the X-ray analyzer-based multi-contrast zooming CT were carried out at the vertical wiggler beamline BL-14B (Ando *et al.*, 1986 ▸) of the Photon Factory (Tsukuba, Japan) with the optics shown in Fig. 3 ▸. The X-rays that are radiated from the vertical wiggler (Yamakawa *et al.*, 1986 ▸) are linearly polarized in the vertical direction, so there is no loss of X-ray intensity due to the polarization effect, even if all optical elements making use of diffraction by crystals are placed in the horizontal plane. Thanks to this advantage, multiple-crystal optics such as ABI can be easily set up in the experimental hutch. The white beam from the vertical wiggler was monochromated at a wavelength of 0.112 nm by a pair of Si(111) crystals diffracting in the horizontal plane. The incident monochromatic beam was collimated and expanded in the horizontal plane by a Si(220) asymmetric crystal (α = 10°, θ_B_ = 16.96°). The beam that was transmitted through the sample was analyzed and magnified in the horizontal direction by a Si(220) asymmetric crystal (α = 14°, θ_B_ = 16.96°). The collimator and the analyzer were placed in a nondispersive (+, −) geometry for maximizing the throughput and angular resolution. Both crystals were made from float-zone silicon ingots, and their surfaces were mechanochemically polished to remove defects and strain fields. The beam diffracted by the analyzer was recorded by a fiber-coupled X-ray CCD camera (Photonic Science X-ray Coolview FDI 40 mm). The pixel size was 46 µm (H) × 46 µm (V) and the number of pixels was 692 (H) × 516 (V).

The energy and ring current at the Photon Factory was *E* = 2.5 GeV and *I* = 450 mA, respectively. The parameters of the electron beam for beamline BL-14 were as follows: σ_*x*_ = 0.53 mm, σ_*y*_ = 0.045 mm, σ_*x*′_ = 0.128 mrad, and σ_*y*′_ = 0.008 mrad. The source-to-sample distance was *L*_s–s_ = 25 m and the sample-to-detector distance was *L*_s–d_ = 0.5 m. Under these experimental conditions, the effect of Fresnel diffraction due to propagation in free space (Cosslett & Nixon, 1951 ▸; Snigirev *et al.*, 1995 ▸) was negligible. This is because the spatial coherence of the incident X-rays at the Photon Factory is not as high as at the third-generation synchrotron radiation facilities.

**Figure 4:**
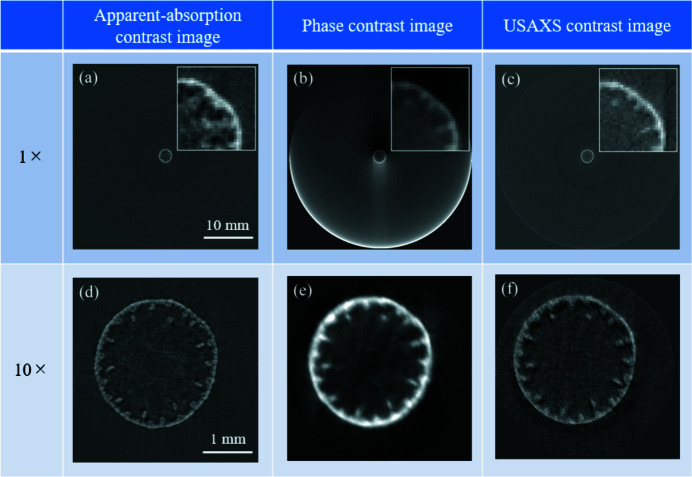
Reconstructed slice images of the stem of Japanese pampas grass (*Miscanthus sinensis*), the diameter of which is about 2.3 mm. The images on the left [(*a*) and (*d*)], center [(*b*) and (*e*)] and right [(*c*) and (*f*)] columns are the apparent-absorption contrast, phase contrast and USAXS contrast images, respectively. The magnification factor is *M* = 1 for the images in the top row [(*a*)–(*c*)], and *M* = 10 for those in the bottom row [(*d*)–(*f*)]. For comparison, the insets in (*a*)–(*c*) show digital zoom (10×) images of the upper-right part of the slice images of the sample.

As a sample, we observed the stem of Japanese pampas grass (*Miscanthus sinensis*), the diameter of which was about 2.3 mm. The analyzer was scanned through the Bragg diffraction condition in 1.25 arcsec steps. The number of measured analyzer angles was 41. At each analyzer angle, the sample was rotated around the vertical axis from 0° to 180° in 1° steps for acquiring the CT data set. The exposure time for each image was 1 s. Dark-frame correction was applied to all images. First, we observed the sample at *M* = 1 (ϕ = 0°), and then we zoomed in by changing the azimuthal angle, ϕ, until we reached the optimal magnification (*M* = 10, ϕ = 90°). Image reconstruction was carried out by following the routine procedures for the filtered back-projection method. The tri-modal contrast cross-sectional images of the sample were successfully obtained as shown in Figs. 4 ▸(*a*)–4(*f*). It can be clearly seen that for each contrast (apparent-absorption, phase and USAXS) the vascular bundles and epidermis are more clearly depicted at *M* = 10 than at *M* = 1.

Table 1 ▸ shows the calculated spatial resolution of the entire optical system, Δ, together with Δ_pd_, 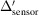 and Δ_pb_. As expected, the spatial resolution is improved by a factor of ten for *M* = 10 compared with *M* = 1. It is also seen that the dominant factor determining the spatial resolution of the entire optical system is the spatial resolution of the area sensor, Δ_sensor_, in our experimental conditions.

**Figure 5:**
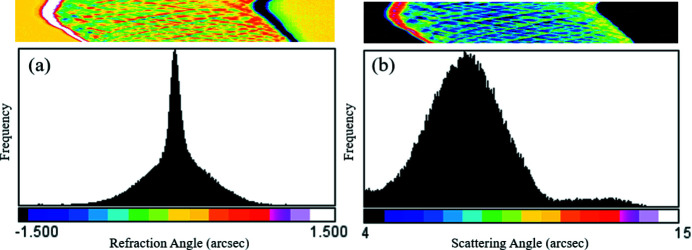
Sinograms (upper) and histograms (lower) of (*a*) the refraction angle, Δθ_cm_, and (*b*) the scattering angle, σ_USAXS_, obtained for *M* = 10.

The angular resolution of the optics affects the quality of the phase contrast and USAXS contrast images, but not the quality of the absorption contrast images. In the analyzer scanning method, the angular resolution depends on the angular width of the rocking curve of the analyzer, 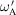 , given by equation (8). In general, the angular resolution deteriorates with the increase in the magnification factor. For example, 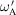 was calculated to be 4.0″ for *M* = 1 (ϕ = 0°) and 11.5″ for *M* = 10 (ϕ = 90°). This shows that, for *M* = 10, the angular resolution is decreased by a factor of about three compared with the case where *M* = 1. Fig. 5 ▸ shows histograms and sinograms of the refraction angle, Δθ_cm_, and the scattering angle, σ_USAXS_, obtained for *M* = 10. The refraction angle, Δθ_cm_, is distributed between −0.8″ and +0.8″, while the scattering angle, σ_USAXS_, is distributed at around 8″. Since the refraction angle, Δθ_cm_, is about ten times smaller than the scattering angle, σ_USAXS_, the refraction (phase) contrast images are more sensitive to 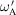 than the USAXS contrast image. This may be the reason why the phase contrast image in Fig. 5 ▸(*e*) looks more blurred than the USAXS contrast image in Fig. 5 ▸(*f*). It is worth noting that, from the histogram of the refraction angle in Fig. 5 ▸(*a*), the angular resolution of the optics is estimated to be better than 0.2″ for *M* = 10. The angular resolution of the optics can be improved, for example, by using a higher-order diffraction for the collimator and the analyzer. Table 2 ▸ shows an example of the calculated ω_s_ for σ-polarized X-rays with a wavelength of 0.112 nm. It can be seen that ω_s_ for Si 333 diffraction is about three times smaller than that for Si 220 diffraction.

By changing the asymmetric angle, α, of the analyzer, the range of the available magnification factor can be extended. For example, assuming an asymmetric angle, α, of 16.7°, the magnification factor, *M*, is calculated to vary between 0.008 (ϕ = −90°) and 122 (ϕ = 90°). This means that the range of available magnification is about twelve times larger. It should be noted, however, that there is a restriction in that the entire sample must always be within the field of view during the sample rotation in CT measurements. If this condition is not met, artifacts will occur and the correct images will not be obtained. In most cases, the range of available magnification is limited by this requirement in CT measurement. This is the reason why we set the magnification factor at *M* = 10 in the present experiments. For observing larger (smaller) samples, the magnification needs to be reduced (increased). We plan to mitigate this limitation by introducing, for example, tomosynthesis (Matsuo *et al.*, 1993 ▸; Dobbins III & Godfrey, 2003 ▸) and lamino­graphy (Helfen *et al.*, 2005 ▸; Hirano, Takahashi *et al.*, 2016 ▸) instead of conventional CT.

In the one-dimensional zooming optical system shown in Fig. 3 ▸, the sample image is not magnified in the direction perpendicular to the plane of diffraction of the Bragg magnifier. This optical system, however, is effective for observing specimens that have almost uniform structures in one direction, such as fibrous objects.

A two-dimensional zooming optical system can also be realized by utilizing a pair of asymmetric crystals arranged in a σ–π configuration. One of the advantages of the 2D zooming optics is that the magnification factor can be independently controlled in two orthogonal directions. According to our previous results on absorption contrast imaging, the throughput was lower at the Photon Factory than at third-generation synchrotron radiation facilities, but it was still practical enough (Hirano *et al.*, 2015 ▸; Hirano, Yamashita *et al.*, 2016 ▸). We expect that the throughput of the 2D zooming optics will be significantly enhanced at state-of-the-art facilities that can produce diffraction-limited X-rays in both the horizontal and vertical directions, such as fourth-generation synchrotron radiation facilities and X-ray free-electron laser facilities.

## 4. Conclusions

In conclusion, we developed X-ray zooming optics for ABI and applied it to multi-contrast computed tomography. In the proof-of-principle experiments performed at the vertical wiggler beamline BL-14B of the Photon Factory, we obtained tri-modal contrast sectional images of the stem of Japanese pampas grass at *M* = 1 (ϕ = 0°) and *M* = 10 (ϕ = 90°) by following the routine procedures for the filtered back-projection method. It was confirmed that for each contrast (apparent-absorption, phase and USAXS), the image quality at *M* = 10 was superior to that at *M* = 1. These results open up new possibilities for observing an entire sample or regions of interest within a sample at optimal magnification, and are expected to be useful for materials science, condensed matter physics, archeology and biomedical science.

## Figures and Tables

**Table 1:** Calculated spatial resolution of the entire optical system, Δ, for *M* = 1 and *M* = 10

*M*	Δ_pd_ (µm)	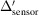 (µm)	Δ_pb_ (µm)	Δ (µm)
1×	2	100	25	103
10×	0.63	10	2.5	10.4

Note: Δ_pd_, 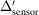 and Δ_pb_ are related to the effects of penetration depth, spatial resolution of the area sensor and penumbral blurring, respectively.

**Table 2:** Example of the calculated ω_s_ for σ-polarized X-rays with a wavelength of 0.112 nm

	Si 111	Si 220	Si 331	Si 422	Si 333	Si 511	Si 440
ω_s_	4.87″	3.58″	1.42″	1.70″	1.11″	1.11″	1.38″
